# Feed-forward neural network assisted by discriminant analysis for the spectroscopic discriminantion of cracked spores *Ganoderma lucidum*: A prospective biotechnology production tool

**DOI:** 10.1186/2191-0855-1-40

**Published:** 2011-11-15

**Authors:** Chee Wei Lim, Sheot Harn Chan, Angelo Visconti

**Affiliations:** 1 Food Safety Laboratory, Applied Sciences Group, Health Sciences Authority, 11 Outram Road, 169078, Singapore; 2 Institute of Sciences of Food Production, National Research Council, Via Amendola, 122/O, 70126 Bari, Italy

**Keywords:** Adulteration, Artificial neural network, Cracked spores, Feed-forward, *Ganoderma*, Spectroscopy

## Abstract

A major problem for manufacturers of cracked spores *Ganoderma lucidum*, a traditional functional food/Chinese medicine (TCM), is to ensure that raw materials are consistent as received from the producer. To address this, a feed-forward artificial neural network (ANN) method assisted by linear discriminant analysis (LDA) and principal component analysis (PCA) was developed for the spectroscopic discrimination of cracked spores of *Ganoderma lucidum *from uncracked spores. 120 samples comprising cracked spores, uncracked spores and concentrate of *Ganoderma lucidum *were analyzed. Differences in the absorption spectra located at ν1 (1143 - 1037 cm^-1^), ν2 (1660 - 1560 cm^-1^), ν3 (1745 - 1716 cm^-1^) and ν4 (2845 - 2798 cm^-1^) were identified by applying fourier transform infra-red (FTIR) spectroscopy and used as variables for discriminant analysis. The utilization of spectra frequencies offered maximum chemical information provided by the absorption spectra. Uncracked spores gave rise to characteristic spectrum that permitted discrimination from its cracked physical state. Parallel application of variables derived from unsupervised LDA/PCA provided useful (feed-forward) information to achieve 100% classification integrity objective in ANN. 100% model validation was obtained by utilizing 30 independent samples. ν1 was used to construct the matrix-matched calibration curve (*n *= 10) based on 4 levels of concentration (20%, 40%, 60% and 80% uncracked spores in cracked spores). A coefficient of correlation (*r*) of 0.97 was obtained. Relative standard deviation (RSD) of 11% was achieved using 100% uncracked spores (*n *= 30). These results demonstrate the feasibility of utilizing a combination of spectroscopy and prospective statistical tools to perform non destructive food quality assessment in a high throughput environment.

## 1. Introduction

Ganoderma lucidum, a fungus commonly known as Lingzhi, has been used as a traditional functional food/medicine for centuries by rulers of the Chinese and Japanese dynasties to achieve enhanced vitality and longevity. These formulae take on exotic forms of special tea and mushroom concoction suitable for daily intake as supplements. Owing to the perceived benefits of these highly desirable medicinal properties localized within the spores (Lin 2001) and further amplified by profit focused producers, commercial demand for *Ganoderma lucidum *outstripped its natural occurrence in nature. Consequently, cultivation techniques were developed to cater for mass production. Some channels used to perform cultivation include horizontal stirred tank reactor and solid state fermentation (Habijanic and Berovic, 2000; Yang et al. 2003; Hsieh and Yang 2004). Both types of cultivation strategies have been reported to yield reasonable fruit bodies suitable for general use. A major problem for manufacturers of cracked spores *Ganoderma lucidum *therefore, is to ensure the raw materials supplied are consistent (Li et al. 2011). According to Recital 11 of the European Union Regulation on the hygience of foodstuffs No 852/2004, the application of hazard analysis and critical control point (HACCP) principles to primary produce is not yet generally feasible (Cerf and Donnat, 2011). By this same principle, rapid methods (practicable in a factory environment) are therefore required to test materials prior to its conversion into the finished product.

The chemical structure of triterpenoids of *Ganoderma lucidum *comprised mainly of ganodermic acid and its alcohol moieties and aldehydes (Sanodiya et al. 2009), among others. Conventional analytical methods applied to characterize these triterpenoids involves the use of liquid chromatography such as reverse-phased high-performance liquid chromatography (HPLC) to separate the complex mixtures and identify them based on their absorbance at 235 nm, 243 nm and 251 nm in methanol using ultra-violet (UV) detector (Chyr and Shiao 1991; Shiao et al. 1989). Post column UV detection at 243 nm was then applied to quantify the triterpenoids. HPLC analysis therefore involves the administration of a series of indirect and irreversible destructive protocols. Common to all industrial food processes, the ability to obtain real-time information via an integrated and non destructive quality control system is an attractive option financially, since process delinquencies due to poor materials control may now be greatly reduced via an intermediate quality assessment step implemented at the raw materials level.

For this purpose, we developed a workflow involving the direct application of rapid FTIR and its feed-forward ANN model to perform classification of cracked spores of *Ganoderma lucidum *originating from a single producer to assess its raw materials (quality) consistency. Cracked spores, uncracked spores and concentrate of *Ganoderma lucidum *were used to construct the model by utilizing their principal frequency bands. PCA and LDA were applied on a 120 sample data pool. The values derived by applying PCA/LDA analyses were then fed into an ANN model constructed using 4 hidden nodes. Model validation was performed using 33% (random) data set.

## Material and methods

### Samples

120 samples comprised mainly of cultivated Ganoderma lucidum strain were analysed. These samples were received in bulk unpackaged powder form. To achieve model integrity, 90 samples comprising reference materials of cracked spores, uncracked spores and concentrate of *Ganoderma lucidum *were used as markers in our model building. To ensure sample uniformity, each sample matrix was homogenized using mortar and pestle prior to performing FTIR analysis.

### FTIR

Spectral measurements were carried out on a Shimadzu FTIR 8400S system equipped with a germanium coated KBr beam splitter, a Michelson type (30° incident angle) interferometer and a temperature controlled high sensitivity detector (DLATGS). Spectra were recorded under diffuse reflectance mode at a resolution of 4 cm^-1 ^set to 128 scans. Spectra were acquired and processed using the as-supplied IRSolution program for microsoft windows. Absorbance peaks were then converted to second derivative using 15 convolution points (Munakata 1998). Calculation was performed by dividing the area of the absorption band of interest with a fixed frequency band located between 810 - 786 cm^-1^. This band was represented to be a silica-related absorption band (Bosch Reig et al. 2002) originating from the sample holder used to perform spectrum acquisition. In this paper, we considered using the area (relative) of each spectrum at pre-defined frequency bands to perform model construction. These frequency bands were represented by ν1 (1143 - 1037 cm^-1^), ν2 (1660 - 1560 cm^-1^), ν3 (1745 - 1716 cm^-1^) and ν4 (2845 - 2798 cm^-1^) respectively.

### Statistics and data processing

#### Principal component analysis (PCA)

A commercially available Windows version of the JMP 9.0 (division of SAS Institute Inc, Cary, North Carolina, USA) was used to perform PCA, LDA and ANN analyses. For PCA analysis, the most prominent directions of the high-dimension data were identified. The dimensionality of the dataset was reduced by first applying a linear combination of the standardized original variables possessing the greatest possible variance, thereby creating the first principal component (PC 1). The second component (PC 2) was created based on the linear combination of the standardized original variables having the greatest possible variance and was uncorrelated with all previous defined components. By this principle, a reduced set of variables was achieved (Meloun et al. 1992). In this paper, unsupervised PCA was performed.

#### Linear discriminant analysis (LDA)

In DA, the classification variable is fixed and predicted by the continuous variables. JMP's implementation permits three types of DA(s) defined by linear, quadratic and mixed linear/quadratic. Briefly, LDA requires the Y variables to be normally distributed with the same variance and covariance but with different means for each group, while quadratic discriminate analysis requires the covariance to be different across the groups. In this paper, we considered applying LDA. To assess the model's classification robustness, 30 independent samples comprising cracked spores *Ganoderma lucidum *were used to perform cross-validation.

#### Artificial neural network (ANN)

ANN analysis was used to assess the classification integrity predicted by the discriminant function, as well as to predict the amount of uncracked/cracked spores content by comparison with a known. Briefly, the TanH activation function was applied using one hidden layer. Values derived by applying PCA/LDA were fed into a hyperbolic tangent function ($\frac{e^{2x}-1}{e^{2x}+1}$) that transforms values between -1 and 1. It is the centred and scaled version of the logistic function, with *x *representing the linear combination of the X variables. No penalty constraint was applied to the method. To predict the approximate crack/uncracked spores content (qualitative assessment) present in a known material, 4 levels of concentration of 20%, 40%, 60% and 80% uncracked spores in cracked spores were prepared.

## Results

### Differences in absorption spectra of Ganoderma lucidum samples

**Figure 1:**
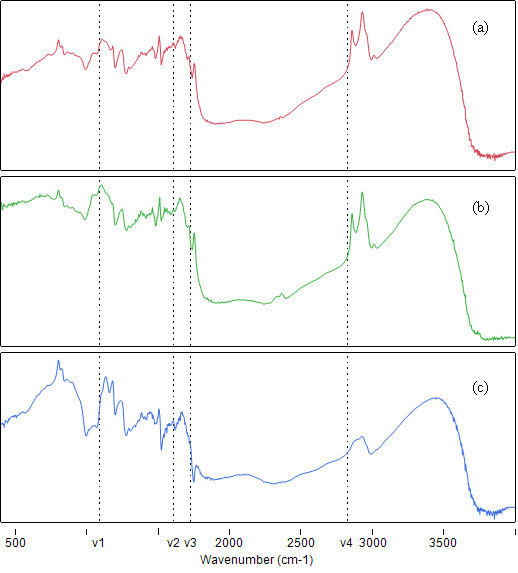
**Area-normalised FTIR absorption spectra of **a **uncracked spores, **b **cracked spores and **c **concentrate of *Ganoderma lucidum***. ν1, ν2, ν3 and ν4 represent the midband frequency of the principal frequency band used to perform PCA/LDA/ANN analysis.

The absorption spectra of the homogenized samples were characterized by feature-rich frequency bands representative of major components of Ganoderma lucidum. These frequency bands are collectively defined by the fingerprints of polysaccharide, polysaccharide-peptide complex, β-glucans, lectins, organic germanium, adenosine, triterpenoids and nucleotides combined (Gao et al. 2004; Mizuno 1995; Liu 1999). Area-normalized spectra of cracked spores, uncracked spores and concentrate of *Ganoderma lucidum *are shown in Figure 1a, b and 1c, respectively. In particular, spectral variations were observed in the region 1050-1800 cm^-1 ^(hetero-oxy and carbonyl containing) and 2700-3000 cm^-1 ^(aliphatic compounds) across all 3 types of reference materials. By using an *F*-test on the ratio of the variances in each dataset (Kemsleyet al. 1994), the null hypothesis that no spectral variation is caused by plant species can be rejected at the 0.10% level. This suggested that spectral variation between cracked spores, uncracked spores and concentrate of *Ganoderma lucidum*, is significant.

### Principal component analysis (PCA), linear discriminant analysis (LDA)

**Figure 2:**
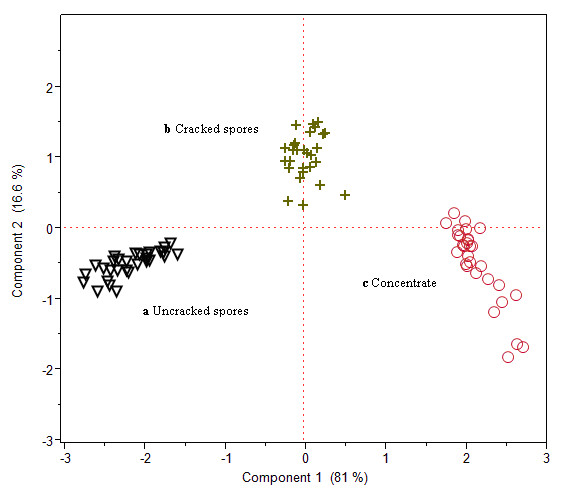
**2-D score plots of PCA results for **a **uncracked spores **b **cracked spores and **c **concentrate of *Ganoderma lucidum *by the first two principal components**.

In order to classify and quantify these variations, linear transformation method of PCA was first applied. 97.6% of the variance of the original dataset was explained by the first two PCs, as shown in the 2-D score plots of PCA results in Figure 2. Frequency band ν1 has the highest weight on the first PC (explaining 81.0% of the variability) while ν2, ν3 and ν4 dominated the second PC (explaining 16.6% of the variability). From Figure 2, 3 distinct clusters representing cracked spores, uncracked spore and concentrate of *Ganoderma lucidum *were obtained, each achieving 100% classification objective. The results suggested that a classification rule based on nearness to group means is appropriate. LDA was then applied and validation performed using 30 independent samples containing pure cracked spores. A summary of the predicted group membership of *Ganoderma *samples is shown in Table 1. From Table 1, it is clear that all 30 samples were classified correctly under the category of cracked spores.

**Table 1:** Classification of uncracked spores (US), cracked spores (CS) and concentrate (C) of *Ganoderma lucidum*, and percentage of observations correctly classified.

			**Predicted group membership**				
			**US**	**CS**	**C**	**CS (Validation)**	**Total**
Original	Count	US	30	0	0	0	30
		CS	0	30	0	0	30
		C	0	0	30	0	30
		CS (Validation)	0	0	0	0	0
	%		100.0	100.0	100.0	0	100.0^b^
Cross-validated	Count	US	30	0	0	0	30
		CS	0	30	0	0	30
		C	0	0	30	0	30
		CS (Validation)	0	0	0	30^a^	30
	%		100.0	100.0	100.0	100	100.0^c^

a. 30 independent samples of cracked spores of *Ganoderma lucidum *were used to perform validation only.

b. 100.0% of original grouped cases were correctly classified.

c. 100.0% of cross-validated grouped cases were correctly classified.

### Artificial neural network (ANN)

Coefficient of correlation (*r*) value of 1.0 (both training and validation sets) was achieved for frequency bands ν1, ν2, ν3 and ν4 suggesting perfect model fit. Similarly, a root mean square error (RMSE) of <0.1% was reported. Clearly, the results reported by the ANN model confirmed the classification outcome obtained by applying PCA/LDA analyses.

## Discussion

While it is possible to improve the first PC score further by reducing the variables (frequency bands), such approach raised some concerns within the framework of spectroscopy. Indeed, while PCA and LDA are useful tools suitably used to extract features that are focused on discriminating between classes via dimension reduction strategy, the error increment due to dimension reduction has to be without sacrificing the discriminative power of classifiers (Benediktsson and Sveinsson 1997). In this work, we did not observe such limitation. Rather, by shrinking the variables pool further, the advantage of utilizing the maximum chemical information provided by the absorption spectra will not be fully tapped. For this purpose, the values obtained for the PCs and canonical functions (LDA) were fed into an ANN model using 4 hidden nodes (33% random data holdback) to ascertain the classification outcome obtained when PCA and LDA were applied.

Within the framework of linear discriminant function, 3 distinct clusters representing cracked spores, uncracked spores and concentrate of *Ganoderma lucidum *were established. In order to better translate the materials quality consistency into quantifiable numbers suitable for routine monitoring purpose, a calibration curve comprising 4 levels of concentration (*n *= 10) at 20% concentration interval of cracked spores (in uncracked spores medium) was prepared. Amongst the 4 frequency bands (ν1, ν2, ν3 and ν4) previously identified for discriminant analyses, only frequency band ν1 provided an acceptable *r *value of 0.97. This observation is in parallel to the ν1 dominated first principal component score prior discussed. Using the uncracked spores as a known concentration measurement criteria (*n *= 30), a mean value of 97% (uncracked spores) was reported based on the calibration curve with a RSD value of about 11%. Using the inverse correlation of cracked spores and uncracked spores, a sample categorized as pure cracked spores would therefore have a corrected composition of about 97 ± 11% cracked spores content. By this preposition, it is possible to translate spectra into qualifiers that can be considered for routine analysis to achieve materials quality consistency monitoring objective (only). While the marriage of PCA/LDA and feed-forward ANN strategies offered potential value to achieve discreet plant-based sample analyses objective, it is also important to consider expanding the model to address disproportionate variance-covariance matrices (Kemsley et al. 1994) of data sets when one cluster became enlarged (on the basis of a known single source supplier).

In brief, this work has examined the use of applying feed-forward ANN assisted by PCA/LDA analyses to discriminate cracked spores, uncracked spores and concentrate of *Ganoderma lucidum *to achieve materials quality consistency monitoring objective. 100% classification integrity was achieved. We found that uncracked spores contained distinctive absorption spectre that can be separated using classical FTIR and its discriminant analysis combined. These results demonstrate the feasibility of utilizing a combination of spectroscopy and prospective statistical tools to perform non destructive food quality assessment in a high throughput environment.

On hindsight, the successful marriage of spectroscopy and its statistical model perhaps lend light to the under-regulated functional food/TCM industry and its processes, towards achieving quality materials supply/control and quality products suitable for a safer public consumption objectives.

## Completing interests

The authors declare that they have no competing interests.
